# Development and Validation of a Terbium-Sensitized LuminescenceAnalytical Method for Deferiprone

**Published:** 2012

**Authors:** Jamshid Manzoori Lashkar, Mohammad Amjadi, Jafar Soleymani, Elnaz Tamizi, Vahid Panahi-Azar, Abolghasem Jouyban

**Affiliations:** a *Department of Analytical Chemistry, Faculty of Chemistry, University of Tabriz, Tabriz, Iran.*; b *Hematology-Oncology Research Center.*; c *Tuberculosis and Lung Disease Research Center.*; d *Liver and Gastrointestinal Diseases Research Center. *^*e*^*Drug-Applied Research Center and *; e *Faculty of Pharmacy, Tabriz University of Medical Sciences, Tabriz, 51664, Iran.*

**Keywords:** Analysis, Terbium-Sensitized, Deferiprone, Tablet, Validation

## Abstract

A sensitive fluorometric method for the determination of deferiprone (DFP) based on the formation of a luminescent complex with Tb^3+^ ions in aqueous solutions is reported. The maximum excitation and emission wavelengths were 295 and 545 nm, respectively. The effects of various factors on the luminescence intensity of the system were investigated and optimized, then under the optimum conditions, the method was validated. The method validation results indicated that the relative intensity at 545 nm has a linear relationship with the concentration of DFP in aqueous solutions at the range of 7.2 × 10^-9^ to 1.4 × 10^-5^ M, the detection and quantification limits were calculated respectively as 6.3 × 10^-9^ and 2.1 × 10^-8^ M, precision and accuracy of the method were lower than 5% and the recovery was between 100.1% and 102.3%. The results indicated that this method was simple, time saving, specific, accurate and precise for the determination of DFP in aqueous solutions. After optimization and validation, the method successfully applied for determination of DFP in tablet dosage forms. The stoichiometry of the Tb^3+^-DFP complex was found as 1:3 and the complex formation constant was 1.6 × 10^16^.

## Introduction

**Figure 1:**
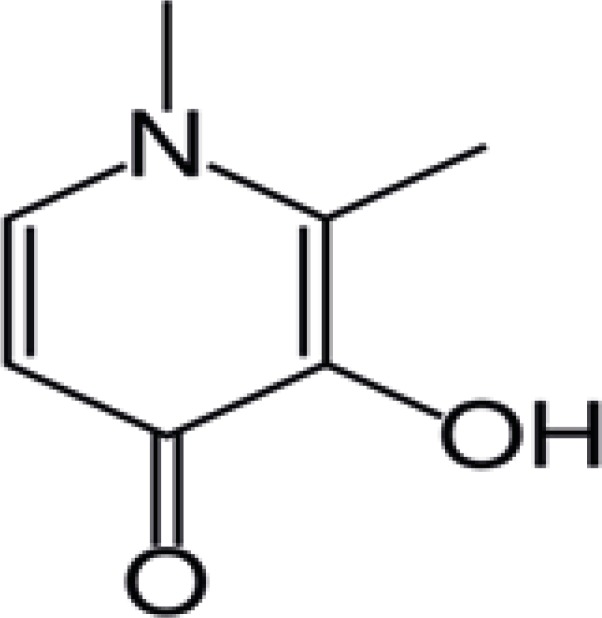
Chemical structure of deferiprone

Deferiprone or 1,2-dimethyl-3-hydroxypyrid-4-one (Figure 1) is an active iron chelator and superoxide radical scavenger which belongs to the new class of chelating agents, *i.e. *alpha-ketohydroxypyridines. Iron overload which can occur as a consequence of chronic transfusion therapy in patients with *β-*thalassemia and sickle cell diseases or due to excessive dietary iron uptake in patients with chronic anemia and hereditary hemochromatosis, causes organ damages and its chelators such as deferiprone (DFP) should be used to remove excess iron from various parts of body (1-5). Intestinal absorption of DFP and one of its analogs was investigated by Taher *et al. *(6). DFP and other chelators could be used to complex cations other than iron which are used for different purposes in biomedical sciences. Zinc complexes of DFP and some related compounds were synthesized and further investigated to improve the bioavailability of zinc after oral administration (7).

Complexes of trivalent lanthanide ions-especially terbium ions (Tb^3+^)- with appropriate ligands have attracted more attention in recent years. Tb^3+^ ions have specific physicochemical properties because of their electronic structure, which is made these ions useful probes and sensors in chemical and biomedical analyses. The electron transition in the 4f shells is responsible for the narrow emission bands in NIR to UV range with long emission lifetimes, large Stock’s shifts (> 200 nm) and μs-luminescence decay times (8-14).

The lanthanide sensitized luminescence is a highly selective method because when lanthanide ions form chelate with certain organic ligands, characteristic emission spectra are obtained. These ions, as analytical reagents, are stable and can form stable luminescent complexes with ligands and have poor absorption in the gastrointestinal tract and cannot penetrate living cells when injected (11). Due to these characteristics, Tb^3+^ ions are widely employed in some applications, such as the investigation of the function of nucleic acids, immunoassays, direct determination of organic compounds and detection of analytes in chromatographic and electrophoretic methods (11-17).

Few methods such as HPLC (4), and voltammetry (3, 18, 19), have been developed for determination of DFP in biological fluids and tablets. In this study, a terbium sensitized luminescence method based on the formation of Tb^3+^-DFP complex in aqueous solution is developed and successfully applied to analyze DFP tablet contents.

## Experimental

*Reagents and solutions*

All chemicals and solvents were of analytical reagent grade. Double distilled water was used throughout. A 10^−2^ M of Tb^3+^ solution was prepared by dissolving the appropriate amount of Tb^3+^ chloride hexahydrate (TbCl_3_.6H_2_O) (Acros Organics, New Jersey, United States) in double distilled water and stored in polyethylene containers to avoid memory effects of terbium adsorbed on glass vessels.

A stock solution (1000 μg/mL) of DFP (Arasto Pharmaceutical Company, Tehran, Iran) was prepared via dissolving in absolute ethanol and stored at room temperature. A 0.01 M Tris-(hydroxymethyl)aminomethan–hydrochloric acid (Tris–HCl) buffer solution was prepared by dissolving a desired amount of Tris–HCl (Merck, Darmstadt, Germany) in water, making up the volume to 100 mL with water. Working standard solutions were prepared daily through dilution of the stock standard solutions with water. DFP (500 mg) tablets were purchased from a local pharmacy store with the trademark of Avicenna Laboratories (Tehran, Iran).

*Apparatus*

Luminescence spectra and intensity measurements were performed by means of a JASCO FP-750 Spectrofluorometer (Tokyo, Japan) equipped with a 150 W xenon lamp and 1.0 cm quarts cells. The excitation and emission monochromator bandwidths were 10 nm. The excitation wavelength was set at 295 nm and the luminescence was measured using the peak height at 545 nm. All measurements were performed at 0 ± 0.1°C using a Peltier thermostated cell holder (Tokyo, Japan). The pH of the solutions was measured with Metrohm model 744 pH meter (Herisau, Switzerland).

*Experimental procedure*

To 10 mL tubes, solutions were added in the following order: 3 mL from 1 × 10^-3^ M Tb^3+^ solution, 0.5 mL from 0.1M buffer solution and suitable aliquots of DFP solution. The mixture was diluted to the mark with double distilled water. The luminescence intensities (F) were measured at λ_ex_/λ_em_= 295/545 nm. All measurements were the average of three replicates.

*Method validation*

The method validation included accuracy, precision, linearity, limit of detection, limit of quantification and stability.

Linearity was evaluated by analyzing standard solutions of DFP in the range of 7.2 × 10^-9^ to 1.4 × 10^-5^ M. The luminescence intensities were plotted versus DFP concentrations in samples and the calibration equation was obtained by linear regression analysis.

The detection and quantification limits calculated as 3S_b_/m and 10 S_b_/m respectively, where S_b_ is standard deviation of 10 blank samples luminescence intensities and m is slope of calibration graph.

The precision of the method was assessed through intra-day and inter-day precisions. For intra-day precision, 6 repeated analyses of standard solutions, at the concentrations of 0.072 × 10^-6^, 7.2 × 10^-6^ and 13.0 × 10^-6^ M performed. Inter-day precision was evaluated by repeated analyses performed on 3 days using the same samples, then relative standard deviations (RSDs) were calculated.

Accuracy was expressed as percent of deviation between the true and measured values and recovery was expressed as a percent of the obtained value to real value of the sample. To assess the accuracy and recovery, replicate analyses of standard solutions, at the concentrations of 0.072 × 10^-6^, 7.2 × 10^-6^ and 13.0 × 10^-6^ M were performed.

For the determination of short term room temperature stability, three samples at the concentrations of 0.072 × 10^-6^, 7.2 × 10^-6^ and 13.0 × 10^-6^ M were prepared and kept at room temperature (23 ± 2 °C) for 12 hours, after this period samples were analyzed, then the samples luminescence intensities were compared with those of the fresh stock solutions. For evaluation of stock solution stability, stock samples were prepared and kept at room temperature for 6 hours, then samples prepared and refrigerated for 7 days, then samples were analyzed and the luminescence intensities were compared with the fresh stock solutions.

*Sample preparation*

Ten DFP tablets were ground into homogenized powder. Then 0.8160 g powder, corresponding to one tablet, was dissolved in about 10 mL of absolute ethanol in a small beaker. The solution was filtered, the residue was washed with ethanol several times, and then diluted with ethanol to 100 mL. Working solution was prepared by appropriate dilution of this sample solution, so that the final concentration was within the linear range.

## Results and Discussion

*Spectral analysis*

*Luminescence spectra*

**Figure 2:**
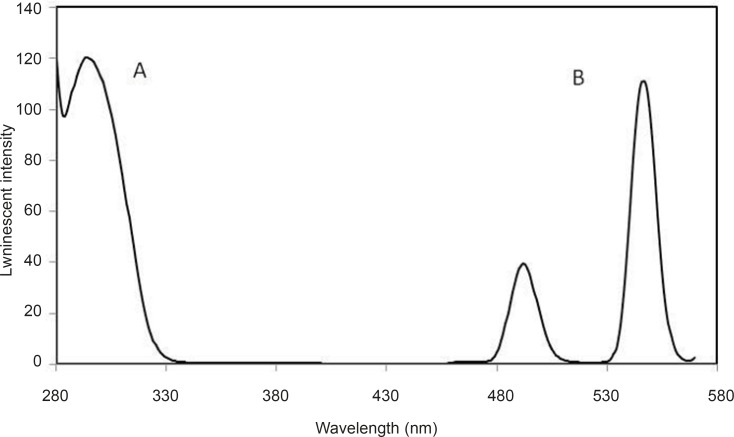
Luminescence excitation (A) and emission (B) spectra of: Tb^3+^-DFP complex. Experimental conditions: [Tb^3+^] = 10^5-^ M; [DFP] = 7.2 × 10 ^-6^ M; pH = 7.5; λ_ex_ /λ_em_=295 nm/545 nm

Luminescence emission and excitation spectra of Tb^3+^–DFP are shown in Figure 2. 

Solutions containing only Tb^3+^ or DFP did not show any measurable luminescence with excitation at 295 nm. Under the same conditions, the characteristic luminescence spectrum of Tb^3+^-DFP was observed, with two emission peaks at 545 nm and 490 nm. These peaks are the characteristic luminescence peaks of Tb^3+^ and correspond to ^5^D_4_→^7^F_6_ and ^5^D_4_→^7^F_5_ transitions, respectively, of which the emission at 545 nm is much stronger. The luminescence intensity was proportional to the concentration of DFP.

*Absorption spectra*

**Figure 3:**
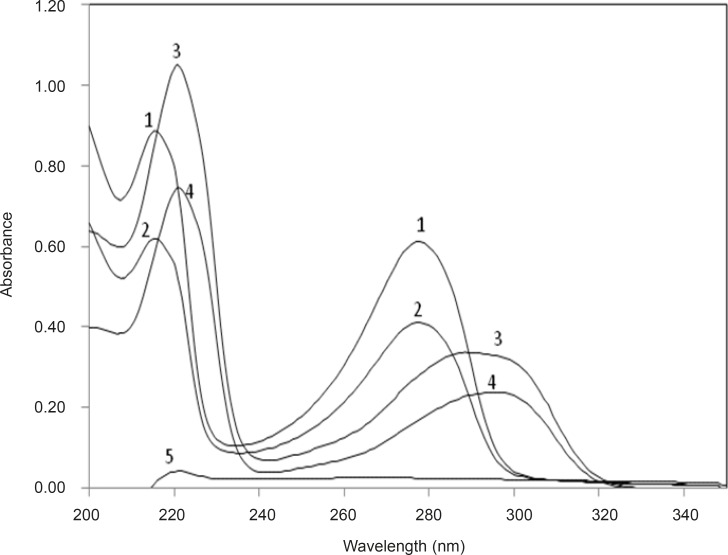
Absorption spectra of DFP in different systems (background correction was done by using a reference solution): DFP (1, 2);.)Tb^3+^-DFP (3, 4) and Tb^3+^(5). Conditions: [Tb^3+^] = 1.0 × 10 ^-4^ M, [DFP] =2.87 × 10 ^-5^ M (1, 3), 1.44 × 10 ^-5^ M (2, 4).

Absorption spectra of Tb^3+^ (spectrum 5, [Tb^3+^]= 10^-4^ M), DFP (spectra 1 and 2, [DFP]=2.85 × 10^-5^ M and [DFP]=2.85 × 10^-5^ M) and Tb^3+^-DFP (spectra 3 and 4, [Tb^3+^]= 10^-4^ M + [DFP]=2.85 × 10^-5^ M and [Tb^3+^]= 10^-4 ^M + [DFP]=1.44 × 10^-5^ M) are shown in Figure 3. It can be seen that after addition of Tb^3+^ into the DFP solution, a small red shift occurred in the maximal absorption peak, which is due to the formation of Tb^3+^-DFP complex.

*Factors affecting the luminescence intensity of the system*

*Effect of pH*

**Figure 4:**
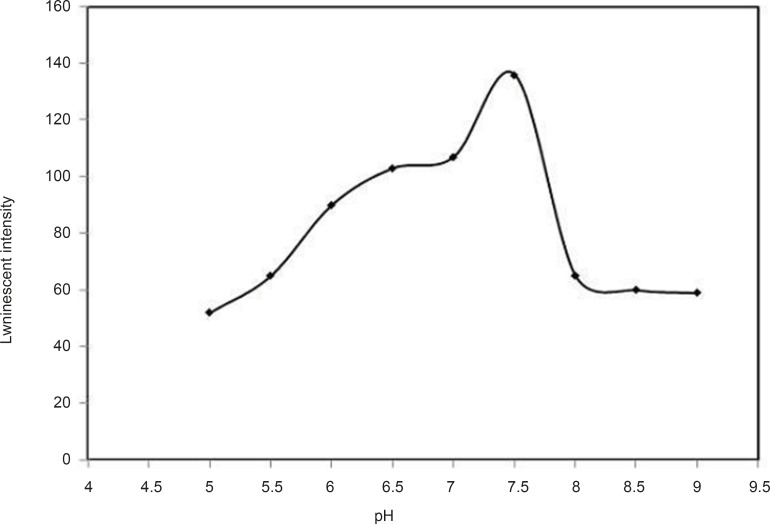
Effect of pH, conditions: [Tb^3+^] =10^-5^ M, [DFP] = 7.2 × 10 ^-6^ M, λ_ex_ /λ_em_=295 nm/545 nm

A series of Tris buffer solutions with different pH values but the same concentrations of other reagents together with corresponding blank solutions were prepared and their luminescence signals were measured at λ_ex_/λ_em_= 295 nm/545 nm. As shown in Figure 4, the luminescence intensity of Tb^3+^–DFP complex strongly depended on pH and reached a maximum value at 7.5. Thus, pH 7.5 was selected for the following experiments.

*Effect of buffer concentration*

**Figure 5:**
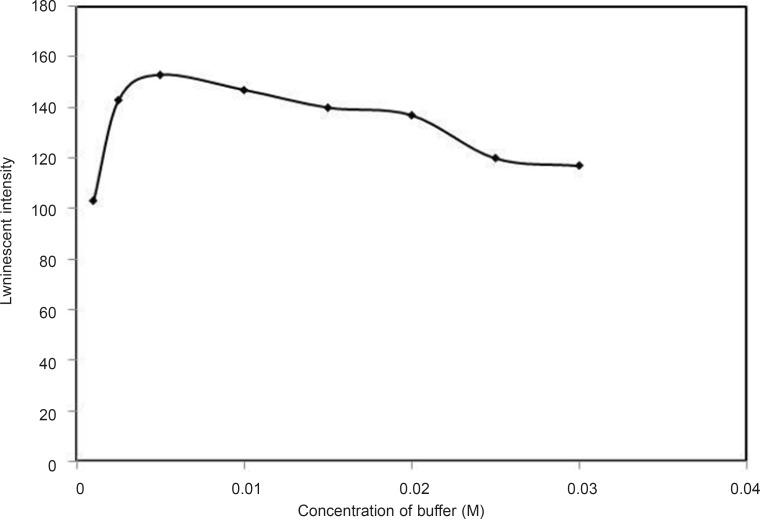
Effect of buffer concentration, conditions: [Tb^3+^] =10^-5^ M, [DFP] = 7.2 × 10 ^-6^ M, pH=7.5.

The effect of different concentrations of buffer on the luminescence intensity of the system is shown in Figure 5. At lower concentrations of Tris, the OH groups of water molecules surrounded the terbium ions and actrd as effective luminescence quenchers due to OH oscillation, thus leading to a decrease in the luminescence intensity. As the concentration of buffer is increased, Tris ligands might prevent Tb^3+^ ion from coordinating water around and so the luminescence intensity is increased. The results indicated that 0.5 mL of 0.1 M Tris–HCl buffer solution in 10 mL mixture was the optimum buffer volume.

*Effect of terbium (III) concentration*

**Figure 6:**
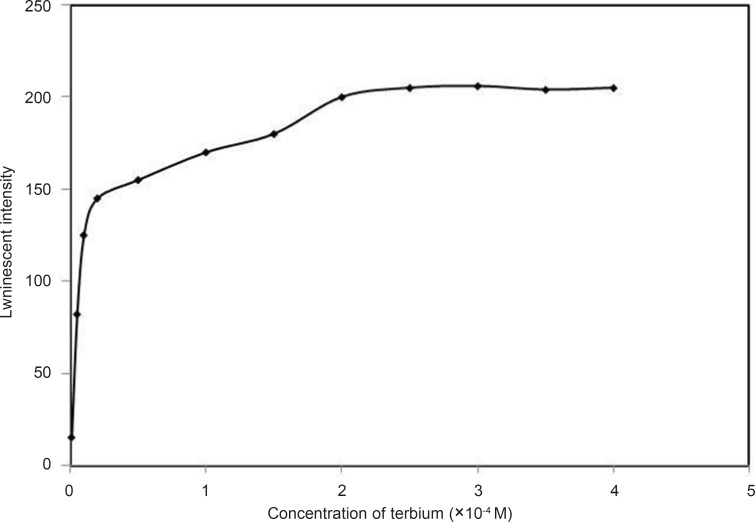
Effect of terbium (III) concentration, conditions: [DFP] = 7.2 × 10 ^-6 ^M, pH = 7.5

The effect of Tb^3+ ^concentration on the luminescence intensity of Tb^3+^–DFP system was studied when the pH and concentration of buffer got fixed at 7.5 and 0.005 M . The results are shown in Figure 6.

**Figure 7:**
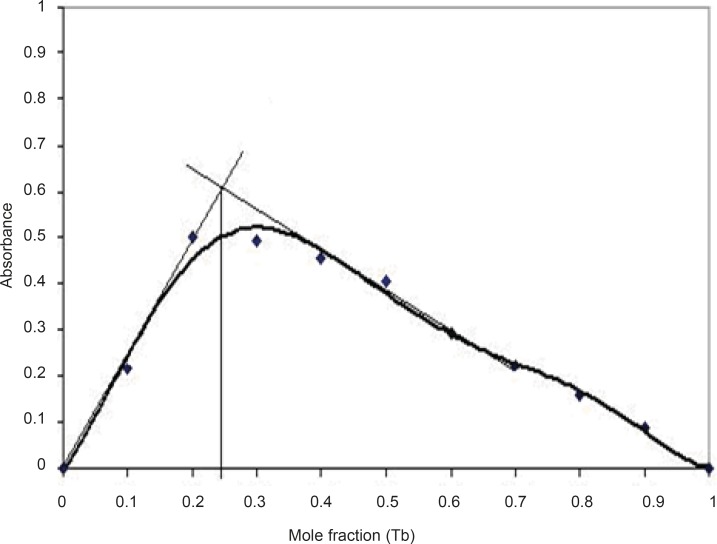
Determination of the stoichiometry of the terbium (III), conditions: DFP complex using Job’s method; [DFP] and [Tb^3+^] = 3.0 × 10^-4^ M, pH=7.5

It can be seen that luminescence intensity was the highest when the concentration of Tb^3+^ in the mixture was 3.0 × 10^-4^ M. Therefore, the concentration of Tb^3+^ in the mixture was chosen at 3.0 × 10^-4^ M for further investigations. The stoichiometry of the complex was studied using Job’s method, i.e. equal concentration of Tb^3+^ and DFP were used. The ratio of molar fraction Tb^3+^: DFP was varied and luminescence intensity of the complex was recorded. The maximum intensity was obtained at a mole fraction of 0.25 (Figure 7), thus a stoichiometry of Tb^3+^: DFP achieved as 1:3. The complex formation constant was calculated using a method described elsewhere (20) and was 1.6 × 10^16^.

*Effect of temperature*

**Figure 8:**
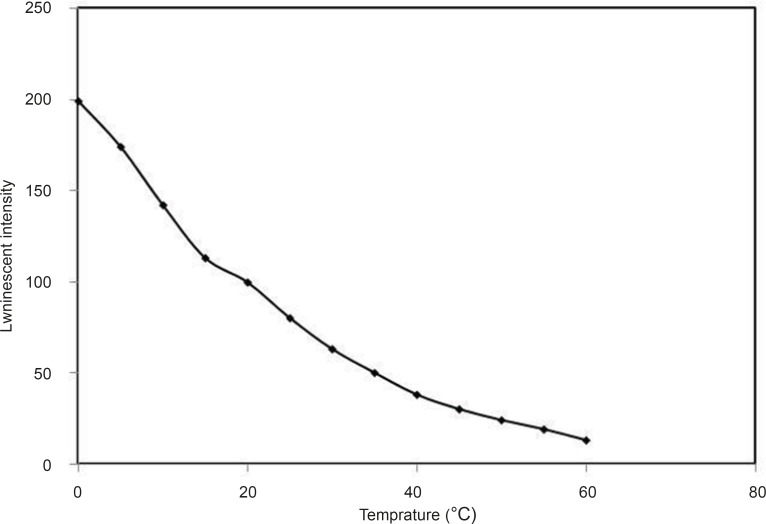
Effect of temperature, conditions: [Tb^3+^] =3 × 10^-4^ M, [DFP] = 7.2 × 10 ^-6^ M, pH = 7.5

Temperature had great influence on the luminescence intensities of this system. The luminescence intensity sharply decreased with temperature from 0°C to 60°C. Therefore, 0°C was selected for further study (Figure 8).

*Effect of time*

**Figure 9:**
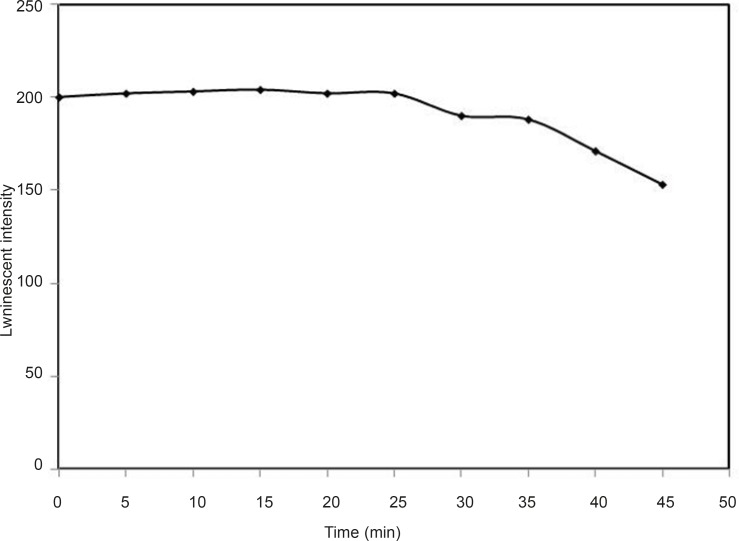
Effect of time, conditions: [Tb^3+^] = 3 × 10^-4^M, [DFP] = 7.2 × 10 ^-6^ M, pH = 7.5

Under the optimum conditions, the effect of time on the luminescence intensity was studied at 0°C. The results showed that the luminescence intensity is stable at the first 25 min after addition of all reagents. In this study, 3 min was set as the optimum value for all luminescence intensity measurements (Figure 9).

*Effect of the order of addition*

Finally, the effect of the order of addition was tested. For this purpose, series of solutions with different addition orders of reagents were measured at λ_ex_/λe_m_ = 295 nm/545 nm. Based on the results, we selected Tb^3+^, Tris_HCl, and DFP as the best order for this assay.

*Analytical figures of merit*

Under the optimal conditions, calibration graph for the determination of DFP was constructed. The linearity parameters for DFP are shown in Table 1. Results of linearity evaluation indicated that, this method was linear in the range of 7.2 × 10^-9^ to 1.4 × 10^-5^ M for determination of DFP in aqueous solutions with a correlation coefficient of 0.999. The detection and quantification limits calculated from calibration graphs, are also given in Table 1.

**Table 1:** Linearity parameters of the method in standard solution

**Data point** ^a^	**Slope**	**Y-intercept**	**r** ^b^	**Range**	**LOD** ^c^	**LOQ** ^c^
13	2.56 × 10^7^	2.413	0.999	7.2 × 10^-9^ to 1.4 × 10^-5^	6.3 × 10^-9^	2.1 × 10^-8^

^a^Data point is the number of concentrations included in the calibration graph.^b^r is the correlation coefficient of calibration graph. ^c^The results were achieved through 6 repeated analyses for each sample

Details of the analytical performances of the previously reported methods and the proposed method for the determination of DFP are summarized in Table 2. Compared with the previous methods, the proposed method is simple and has relatively lower detection limit and lower linearity range.

**Table 2:** Comparison of the proposed method with other methods used for determination of deferiprone.

**Method**	**Linear range**	**Detection limit**	**Reference**
**HPLC**	3.5 × 10^-6^-1.4 × 10^-5^	3.5 × 10^-7^	4
**Voltammetry**	9.9 × 10^-5^-5.3 × 10^-4^	1.9 × 10^-5^	19
**Cyclic voltammetry**	3.0 × 10^-5^-1.0 × 10^-3^	1.4 × 10^-5^	3
**Cyclic voltammetry**	5.0 × 10^-5^-1.0 × 10^-3^	5.3 × 10^-7^	18
**Terbium sensitized luminescence**	7.1 10^-9^-1.4 × 10^-5^	6.3 × 10^-9^	This work

The results for precision, accuracy and recovery of the method respectively were given in Tables 3 and 4; these results illustrated that the proposed method is accurate and precise for determination of DFP.

**Table 3:** Precision (RSD %) of the method obtained for standard solutions

**Concentration (×10** ^-6^ **)**					
**0.072**		**7.20**		**13.0**	
**Intra-day**	**Inter-day**	**Intra-day**	**Inter-day**	**Intra-day**	**Inter-day**
**2.9**	4.8	2.1	1.3	1.2	1.3

The precision results achieved by 6 repeated analyses for each concentration

**Table 4:** Accuracy and recovery of the developed method in standard solutions

**Accuracy**			**Recovery**		
**Concentration (×10 ** ^-6^ ** M)**					
0.072	7.20	13.0	0.072	7.20	13.0
4.80	1.25	1.31	100.3	100.1	102.3

The accuracy and recovery results achieved by 6 repeated analyses for each concentration

The stability data for the proposed method was shown in Table 5 and the results indicated that, it is stable in short term room temperature and in stock solutions. 

**Table 5:** Details of the stability of the proposed method for three concentrations investigated

**Short term room temperature stability**				**Stock solution stability**			
**Concentration (×10 ** ^-6^ ** M)**	0.072	7.20	13.0		0.072	7.20	13.0
**Recovery %**	98.9	100.6	103.1		93.0	98.6	101.0
**RSD %**	3.5	2.3	1.2		4.4	1.7	1.2

The results achieved by 6 repeated analyses for each sample

*Analytical application*

*Determination of DFP in tablets*

The developed method was applied to the determination of DFP in tablets prepared according to the sample preparation procedure. For the assay of DFP, the samples must be diluted properly within the linear range of the determination of DFP and the sample solution was analyzed , using the standard calibration method. Label claim of each DFP tablet is 500 mg, and the measured amount of DFP in tablet was 503.3 ± 2.7 mg. The relative standard deviation for determination of DFP in tablets was 2.2% and the obtained recovery was 97.3-102.7%.

## Conclusion

In this study, a new spectrofluorimetric method was developed for the determination of trace amounts of DFP. DFP which can form a complex with Tb^3+^ and remarkably enhance its luminescence intensity at 545 nm. The enhanced luminescence intensity is proportional to the concentration of DFP. The results illustrated that, the proposed method was linear, accurate, precise and stable for determination of DFP in aqueous solutions. This method has been successfully applied to the determination of DFP in tablet samples. In addition to lower LOD of the proposed method in comparison with the HPLC and voltammetric methods, its simplicity could be considered as its main advantage. It requires just simple dilution of the sample and no further pre-treatment is required using the proposed method which is an important parameter especially in the routine application of the method where huge number of samples should be analyzed every day. This is not the case for the previously reported analytical methods.
